# Tianeptine, but not fluoxetine, decreases avoidant behavior in a mouse model of early developmental exposure to fluoxetine

**DOI:** 10.1038/s41598-021-02074-9

**Published:** 2021-11-24

**Authors:** Elizabeth A. Pekarskaya, Emma S. Holt, Jay A. Gingrich, Mark S. Ansorge, Jonathan A. Javitch, Sarah E. Canetta

**Affiliations:** 1 Department of Neuroscience, Columbia University Vagelos College of Physicians and Surgeons, New York, NY USA; 2 Division of Molecular Therapeutics, Department of Psychiatry, Columbia University Vagelos College of Physicians and Surgeons and the New York State Psychiatric Institute, New York, NY USA; 3 Division of Developmental Neuroscience, Department of Psychiatry, Columbia University Vagelos College of Physicians and Surgeons and the New York State Psychiatric Institute, New York, NY USA; 4 Sackler Institute for Developmental Psychobiology, Columbia University Vagelos College of Physicians and Surgeons and the New York State Psychiatric Institute, New York, NY USA; 5 Department of Molecular Pharmacology and Therapeutics, Columbia University Vagelos College of Physicians and Surgeons, New York, NY USA

**Keywords:** Disease model, Anxiety, Depression

## Abstract

Depression and anxiety, two of the most common mental health disorders, share common symptoms and treatments. Most pharmacological agents available to treat these disorders target monoamine systems. Currently, finding the most effective treatment for an individual is a process of trial and error. To better understand how disease etiology may predict treatment response, we studied mice exposed *developmentally* to the selective serotonin reuptake inhibitor (SSRI) fluoxetine (FLX). These mice show the murine equivalent of anxiety- and depression-like symptoms in adulthood and here we report that these mice are also behaviorally resistant to the antidepressant-like effects of adult SSRI administration. We investigated whether tianeptine (TIA), which exerts its therapeutic effects through agonism of the mu-opioid receptor instead of targeting monoaminergic systems, would be more effective in this model. We found that C57BL/6J pups exposed to FLX from postnatal day 2 to 11 (PNFLX, the mouse equivalent in terms of brain development to the human third trimester) showed increased avoidant behaviors as adults that failed to improve, or were even exacerbated, by chronic SSRI treatment. By contrast, avoidant behaviors in these same mice were drastically improved following chronic treatment with TIA. Overall, this demonstrates that TIA may be a promising alternative treatment for patients that fail to respond to typical antidepressants, especially in patients whose serotonergic system has been altered by in utero exposure to SSRIs.

## Introduction

Depression and anxiety-related disorders are the two most common mental health conditions and are in the top ten of all health conditions contributing to disability globally^1^. The etiology of these disorders is highly complex. Genetics, early life adversity, acute stress, traumatic events, hormones, and other factors have all been shown to increase risk^2^. While depression and anxiety-related disorders are distinguished diagnostically, they share considerable overlap in symptomatology, including anhedonia and avoidant behaviors^3,4^. In addition, both conditions are highly comorbid, implying some shared neurobiological substrates.

Treatments for these conditions also often overlap, with nearly all focused on manipulating monoaminergic systems such as serotonin, norepinephrine, and dopamine. Frontline pharmacological treatments for both depression and anxiety disorders are selective serotonin reuptake inhibitors (SSRIs) such as fluoxetine (FLX)^3^. SSRIs function by blocking the serotonin transporter, thereby increasing the amount of available extracellular serotonin. Over a period of weeks, in responsive patients, the resulting increase in serotonin is thought to help normalize mood, feelings of worthlessness, sleep disruption, and anxiety^5^. Unfortunately, about half of patients show no response, and two-thirds fail to fully remit following treatment with frontline SSRIs^6^, with some experiencing a worsening of symptoms and an increased risk of suicide. Therefore, a major unmet clinical need is the ability to identify patients with differing underlying neurobiology for whom treatments with an alternative pharmacological target might be more effective.

Studies in mice can help us better understand how disease etiology may predict treatment response. In mice, avoidant, anhedonic, motivation, and despair-related behavioral measures are used as a proxy for anxiety- and depression-like behaviors in humans^7^. Early exposure to FLX in a sensitive period that corresponds approximately to the third trimester in humans, postnatal day 2–11 in mice, results in increased avoidant behavior and decreased novelty-induced exploration and hedonic drive^8,9^. Intriguingly, in the current study, we find that these avoidant symptoms are not responsive to subsequent SSRI administration in adulthood. These findings may have implications for the children of those who take SSRIs while pregnant. Indeed, a recent longitudinal cohort study in Finland found that rates of depression in the children of women who took antidepressants during pregnancy were significantly elevated compared to the children of women who had depression but never took an antidepressant or discontinued its use during pregnancy^10^. Given the prevalence of anxiety and depression, and the widespread usage of SSRIs as a treatment for these conditions, it is of substantial clinical relevance to understand if prenatal exposure to SSRIs might be an important aspect of medical history that would predict treatment response. In particular, one hypothesis is that this patient subpopulation may benefit from antidepressants that do not directly target serotonergic neurons via autoreceptors or transporters^11,12^.

The atypical antidepressant tianeptine (TIA) was recently shown in preclinical studies to exert its effects on affective behaviors via activation of the mu-opioid receptor (MOR)^13,14^. Although MOR activation is responsible for both the analgesic and addictive effects of opioid pain relievers such as morphine, at clinical doses TIA does not promote euphoria^15^ and has been considered as a viable alternative to frontline treatments that directly target monoaminergic systems for treatment-resistant depression^16^.

Based on the different hypothesized mechanisms of action between TIA and SSRIs, the goal of this study was to investigate the effectiveness of TIA at relieving avoidant behaviors associated with developmental exposure to FLX in mice. To test this, we injected C57BL/6J pups with FLX from postnatal day 2 to postnatal day 11 (P2–P11), and then administered FLX or TIA chronically in adulthood, and evaluated the efficacy of each compound on normalizing avoidant behavior in the open field and novelty suppressed feeding task. Overall, our study is designed to assess and compare the efficacy of FLX versus TIA in a mouse model of gestational SSRI exposure, which may have broader implications for affective disorder subtypes with a shared etiology that are resistant to SSRI treatment.

## Methods

### Subjects

Male and female C57BL/6J (C57) mice (Jackson Laboratories, Bar Harbor, ME) were used to breed experimental pups (32 m, 38 f.). Within each litter, pups were divided into PNFLX (39 total; 18 m, 21 f.) and PNVEH (31 total; 14 m, 17 f.) groups. Pups were weaned at postnatal day 21, and both PNFLX and PNVEH mice were housed in each cage. After postnatal day 90, cages were pseudo-randomly sorted into chronic adulthood administration groups, first FLX or VEH, and then subsequently for TIA or VEH. Two females were removed from the rest of the experiment after baseline phenotyping due to potential health concerns. For the FLX administration experiments, 20 PNFLX and 16 PNVEH mice received FLX in adulthood, and 19 PNFLX and 15 PNVEH mice received VEH. For the TIA administration experiments, 20 PNFLX and 16 PNVEH received TIA in adulthood, and 19 PNFLX and 15 PNVEH received VEH. We analyzed all measures for significant interaction effects with sex, and when no interaction was found sexes were combined for analysis.

129SvEv (129) mice were purchased from Taconic (Taconic Farms, Germantown, New York, USA). Of 57 129SvEv PNFLX mice, 30 received FLX as adults, and 27 received VEH (sexes combined). Of 68 129SvEv PNVEH mice, 32 received FLX, and 36 received VEH. 17 animals did not receive any postnatal administration and are referred to as naïve. Of these mice, 8 received FLX and 9 received the VEH. 129 mice were not treated with TIA. Mice were group-housed 3–5 to a cage, kept on a 12-h light/dark cycle, and given ad libitum food and water unless otherwise noted. All behavioral assays were performed in the light cycle. All procedures were carried out in accordance with guidelines approved by the Institutional Animal Care and Use Committees at Columbia University and the New York State Psychiatric Institute. All data are reported in accordance with the ARRIVE guidelines.

### Drug administrations

#### Postnatal fluoxetine

From P2-P11, pups were given either 10 mg/kg (2 mg/ml) Fluoxetine HCL (Anawa, Kloten, Switzerland) (FLX) or a 0.9% sterile saline vehicle through intraperitoneal injection once per day.

#### Adulthood fluoxetine

Cages with co-housed PNFLX and PNVEH mice were pseudorandomly organized into adult administration groups. FLX was administered through the drinking water for three weeks once all the animals had matured beyond P90. Behavioral testing began after three weeks of administration, and FLX administration continued during testing. The 18 mg/kg solution appropriate for C57 mice^17,18^ was prepared by dissolving FLX into drinking water in opaque water bottles. A 10 mg/kg solution was used for 129 mice^19^. The respective vehicle was water alone. Bottles were changed and weighed every 2–3 days to track consumption.

#### Tianeptine

A two-week washout period followed the completion of post-FLX behavioral testing before beginning TIA administration. Cages were pseudorandomly reorganized into TIA and VEH groups, ensuring distribution of those who had received FLX or VEH during the adult administration period. A 30 mg/kg solution of tianeptine NaCl (Qingdao Sigma Chemical Co., purity verified by NMR analysis) (TIA) was delivered at a dose of 0.1 ml/10 g mouse weight via intraperitoneal injection twice per day for 14 days. TIA was dissolved in 0.9% sterile saline and stored at room temperature for up to three days. The respective vehicle was 0.9% sterile saline.

### Behavior

#### Open field test (OF)

Mice were placed in 42 × 42 × 38 cm plexiglass enclosures for 60 min. Activity was measured using MotorMonitor software (Kinder Scientific, Poway, CA), which recognizes infrared beam breaks in the enclosure. Ambulatory movements, rearing instances, and time spent in the center were analyzed. The center was defined as a central 21 × 21 cm grid. Each of these variables was summed over the first 10 min or total 60 min.

#### Forced swim test (FST)

Mice were habituated to the experimental room for 1 h prior to testing. Mice swam for 6 min in containers filled with 2000 ml of 25–30 °C tap water. Temperature of the water was monitored and the water was changed as needed. Video was recorded and the first 2 min were analyzed for immobility using Videotrack software (ViewPoint, France).

#### Novelty suppressed feeding (NSF)

Mice were food deprived 15 h prior to the beginning of the test, which was performed as previously detailed^20^. A pellet of food was secured onto a white surface and placed in the middle of familiar bedding in a novel arena under 1200 lux overhead lighting. C57 mice were allowed up to 6 min, and 129 mice up to 10 min, to explore the arena and bite the food pellet (recorded as latency to feed). This was followed immediately by 5 min in the home cage, during which mice were presented with a food pellet. Both home cage latency to feed and amount of food consumed were recorded by the experimenter.

### Analysis

Unless otherwise noted, data were analyzed using Prism (GraphPad, San Diego, CA) or using custom scripts in MATLAB (MathWorks, Natick, MA). Data from software that monitored activity during behavioral tests were imported into these programs to be analyzed by experimental group. We performed two-way ANOVAs for all measures to evaluate possible interaction effects between sex and administration. If an interaction was not found, sexes were combined for statistical analysis.

## Results

### C57 mice exposed to fluoxetine from P2-11 exhibit increased avoidant behavior in adulthood

**Figure 1:**
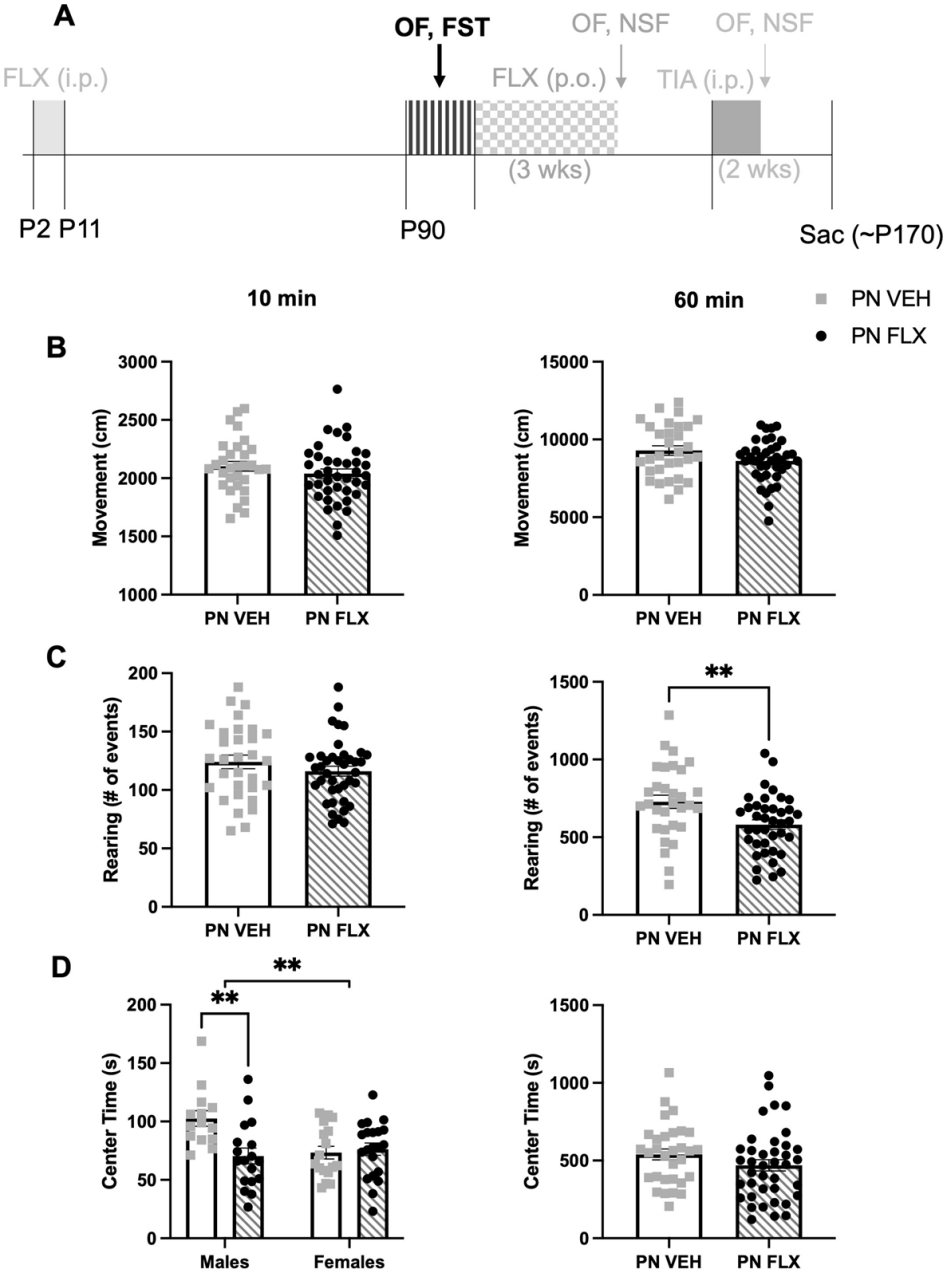
Decreased novelty-induced exploration and increased avoidant behavior in the first 10 and full 60 min of open field following chronic postnatal fluoxetine exposure in C57 mice. (**A**) Timeline of experiment: The window for developmental administration of fluoxetine and future phases of the experiment are illustrated in faded light grey; Black and grey stripes indicate when baseline adult phenotyping using the OF and FST occurred. (**B**) Ambulatory movements in the first 10 min of the open field (left panel, unpaired, two-tailed, t-test, *p* = 0.2846). Same for total 60 min (right panel, unpaired, two-tailed, t-test, *p* = 0.0712). (**C**) Rearing movements in the first 10 min (left, unpaired two-tailed, t-test, *p* = 0.2649). Same for total 60 min (right, unpaired, two-tailed, t-test, **p = 0.0054). (**D**) Time spent in center in the first 10 min (left panel, two-way ANOVA, F(1, 66) = 8.374, **p = 0.0052 for interaction of sex and treatment, F(1, 66) = 3.739, *p* = 0.0574 for main effect of sex, F(1, 66) = 5.812, *p = 0.0187 for main effect of treatment; Bonferroni post-hoc for males: **p = 0.0012, n = 14 PNVEH, 18 PNFLX; for females: *p* > 0.99, n = 17 PNVEH, 21 PNFLX). Same for total 60 min (right panel, unpaired, two-tailed, t-test, *p* = 0.1813). Light gray squares represent PNVEH animals, n = 31. Black circles and grey stripes represent PNFLX animals, n = 39. Interaction effects from the 2-way ANOVA are represented as significance lines between males and females, and the Bonferroni post-hoc results are depicted as significance lines within the males. Bars represent mean. Error bars represent standard error. *p < 0.05, **p < 0.01, ***p < 0.001, ****p < 0.0001.

During P2-P11, we administered either FLX (PNFLX) or VEH (PNVEH) in C57 mice. Adult PNVEH and PNFLX mice underwent behavioral assessment to determine if they had a baseline affective phenotype prior to adult antidepressant treatment (Fig. 1A). We expected to observe decreased novelty-induced exploration and increased avoidant behaviors and behavioral despair, consistent with prior studies in 129 mice^8,9^. We examined the effects of sex and report any interactions accordingly. If no interaction of sex and postnatal exposure were seen, the data from both sexes were combined.

In the open field test (OF), rearing movements were analyzed to measure novelty-induced exploration^21^, and time spent in the center was analyzed to measure avoidant behavior. We separately assessed these behaviors in the first 10 min of the task, the time period most sensitive to changes in avoidant behavior and exploration^22^, as well as the full 60 min. In the first 10 min, PNFLX mice did not rear less than PNVEH mice (Fig. 1C (left panel); unpaired, two-tailed, t-test, *p* = 0.2649, n = 31 PNVEH, 39 PNFLX). However, in the total 60 min, PNFLX mice reared significantly less than PNVEH mice (Fig. 1C (right panel); unpaired t-test, **p = 0.0054, n = 31 PNVEH, 39 PNFLX), consistent with what was previously observed in 129 mice^8^. This suggests that postnatal FLX exposure reduced novelty-induced exploration in C57 mice.

The only interaction between sex and postnatal exposure was found in our measure of time spent in the center in the first 10 min of the open field task. Male PNFLX mice spent less time in the center during the first 10 min compared to their PNVEH counterparts (Fig. 1D (left panel); two-way ANOVA, F(1, 66) = 8.374, **p = 0.0052 for interaction of sex and treatment, F(1, 66) = 3.739, *p* = 0.0574 for main effect of sex, F(1, 66) = 5.812, *p = 0.0187 for main effect of treatment; Bonferroni post-hoc for males: **p = 0.0012, n = 14 PNVEH, 18 PNFLX; for females: *p* > 0.99, n = 17 PNVEH, 21 PNFLX).

Total ambulatory movements in the OF were measured to assess overall locomotion and ensure that other changes in behavior were not the result of broader locomotor dysfunction. PNFLX and PNVEH mice did not differ in their number of ambulatory movements either in the first 10 min (Fig. 1B (left panel); unpaired t-test, *p* = 0.2846, n = 31 PNVEH, 39 PNFLX) or during the total 60 min (Fig. 1B (right panel); unpaired t-test, *p* = 0.0712, n = 31 PNVEH, 39 PNFLX). As overall ambulation did not differ between the groups, the decreases in rearing and center time suggest that postnatal FLX exposure resulted in increased avoidant behavior in male C57s and decreased novelty-induced exploration in both sexes.

The forced swim test (FST) measures behavioral despair in mice and a high percentage of time spent immobile is considered an indicator of depressive-like behavior^22,23^. Time spent immobile was equivalent between PNFLX mice and PNVEH mice (unpaired t-test, *p* = 0.4645, n = 31 PNVEH, 39 PNFLX) suggesting that postnatal FLX did not produce a depressive phenotype in C57 mice. This contrasts with what has been observed in 129 mice^8^ but is similar to what has been seen in P4-P21 PNFLX C57 mice^24^.

Overall, early postnatal FLX exposure in C57 mice produces a persistent behavioral phenotype in adults consistent with decreased novelty-induced exploration and increased avoidant behavior, which is not due to gross abnormalities in locomotor function.

### Chronic fluoxetine exacerbates avoidant behavior in adult mice postnatally exposed to fluoxetine

**Figure 2:**
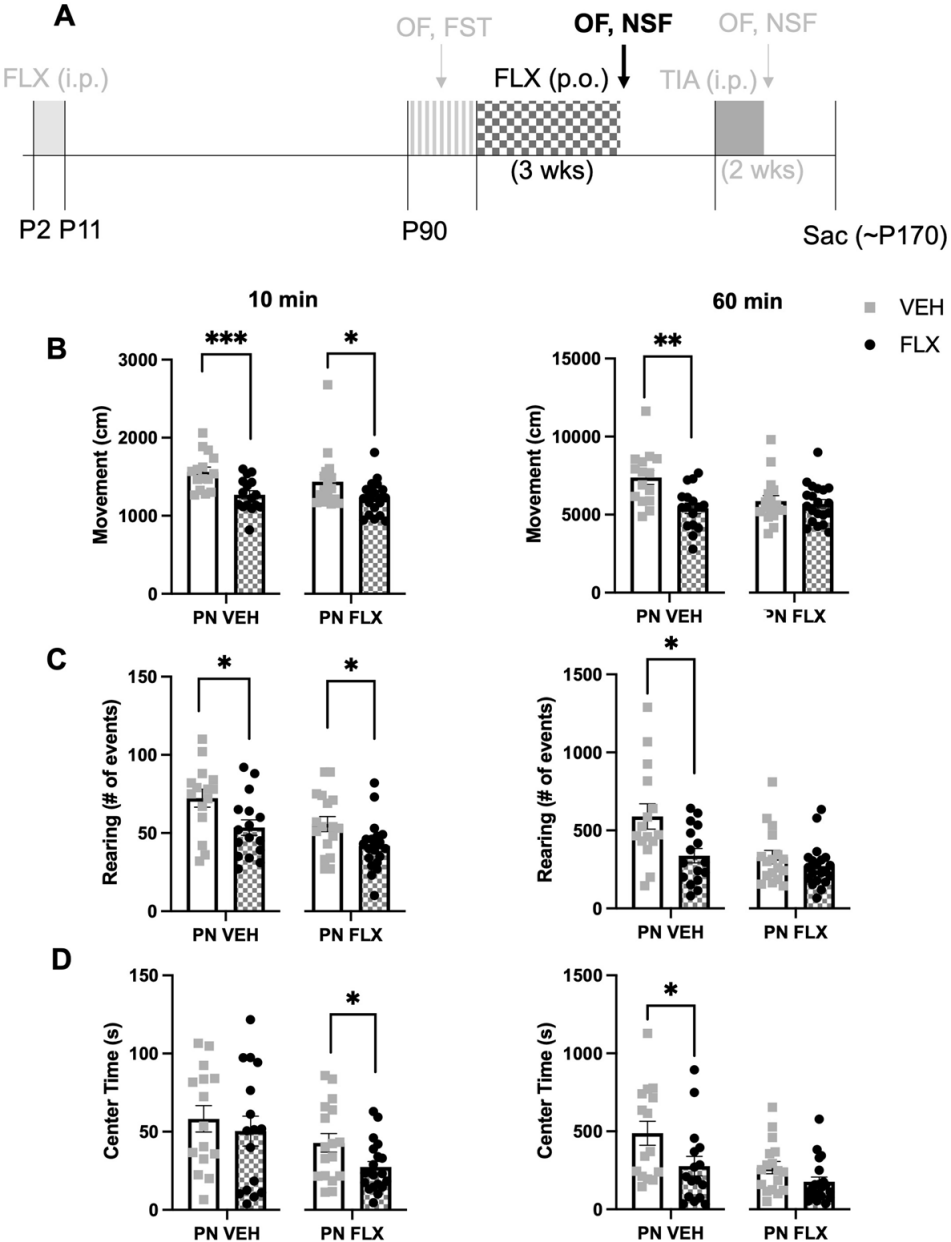
Decreased movement following chronic adult fluoxetine treatment in C57 PNVEH mice and increased avoidant behavior in the first 10 and full 60 min of open field following chronic adult fluoxetine treatment. (**A**) Timeline of experiment: The window for developmental administration of fluoxetine as well as prior phenotyping and future experiments are in faded light grey; Black and checkered grey indicate chronic fluoxetine administration in adulthood and follow up NSF and OF tests. (**B**) Ambulatory movements in the first 10 min of open field (left panel; PNVEH: unpaired t-test, ***p = 0.001; PNFLX: unpaired t-test, *p = 0.044). Same for total 60 min (right panel; PNVEH: unpaired t-test, **p = 0.0014; PNFLX: unpaired t-test, *p* = 0.7012) (**C**) Rearing movements in the first 10 min (left panel; PNVEH: unpaired t-test, *p = 0.0188; PNFLX: unpaired t-test, *p = 0.0224). For total 60 min (right panel; PNVEH: unpaired t-test, *p = 0.0103; PNFLX: unpaired t-test, *p* = 0.2847) (**D**) Time spent in center in the first 10 min (left panel; PNVEH: unpaired t-test, *p* = 0.5455; PNFLX: unpaired t-test, *p = 0.0268). For total 60 min (right panel; PNVEH: unpaired t-test, *p = 0.0418; PNFLX: unpaired t-test, *p* = 0.0745). Light gray squares represent adult vehicle-treated animals. Black circles and checkered bars represent adult fluoxetine-treated animals. N = 15 PNVEH-VEH, 16 PNVEH-FLX, 17 PNFLX-VEH, 20 PNFLX-FLX Bars represent mean. Error bars represent standard error. *p < 0.05, **p < 0.01, ***p < 0.001, ****p < 0.0001.

**Figure 3:**
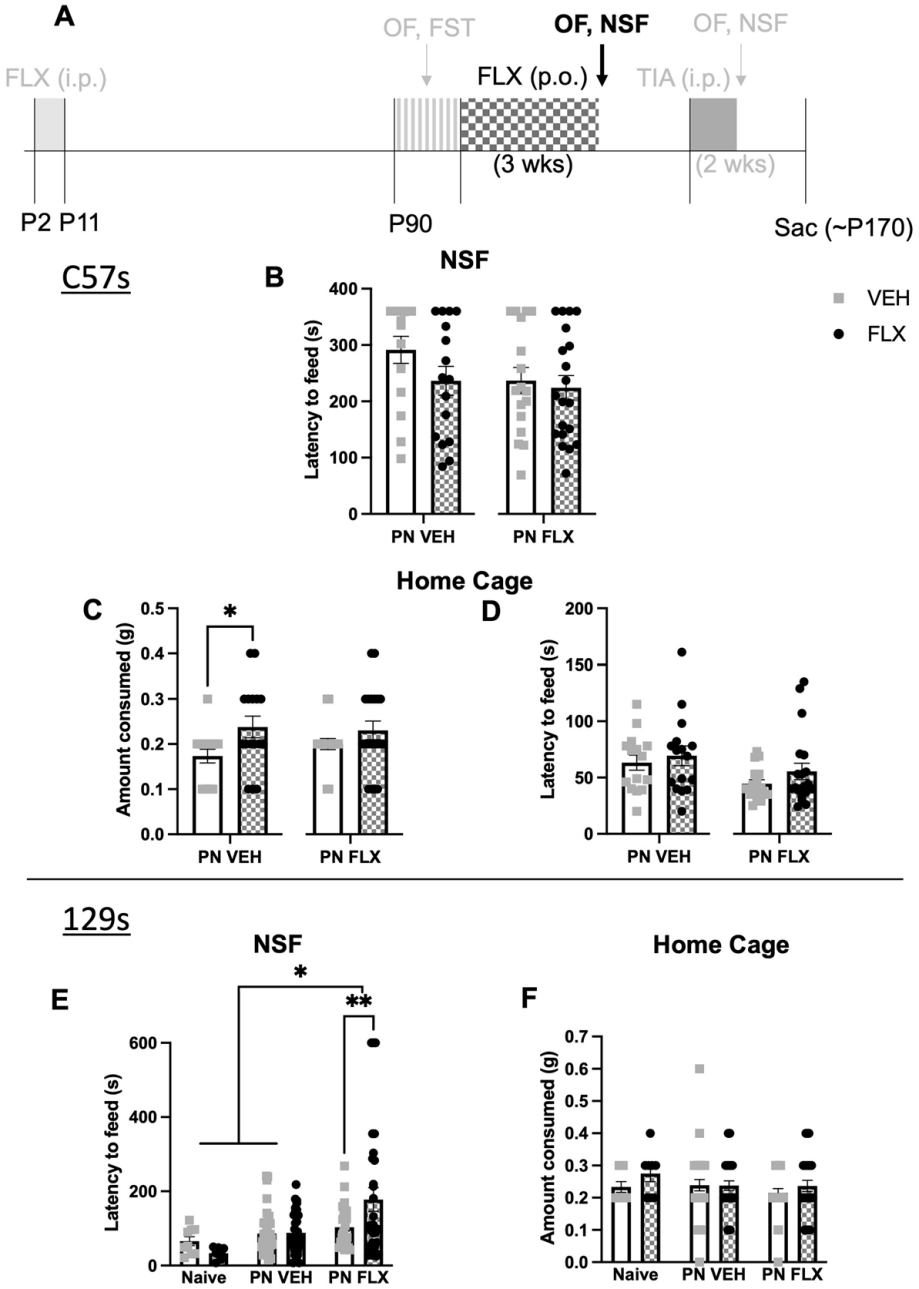
No effect on NSF behavior following adult fluoxetine treatment in C57 PN-FLX mice*,* and increased latency to feed in the NSF following adult fluoxetine treatment in 129 PN-FLX mice. (**A**) Timeline of experiment: The window for developmental administration of fluoxetine, as well as prior phenotyping and future experiments are in faded light grey; Black and checkered gray indicate chronic fluoxetine administration in adulthood and follow up NSF and OF tests. (**B**) Latency to feed in the NSF in C57 mice (PNVEH: unpaired t-test, *p* = 0.1314; PNFLX: unpaired t-test, *p* = 0.6949). (**C**) Home cage consumption in C57 mice (PNVEH: unpaired t-test, *p = 0.0341; PNFLX: unpaired t-test, *p* = 0.2393) (**D**) Latency to feed in the home cage in C57 mice (PNVEH: unpaired t-test, p = 0.5860; PNFLX: unpaired t-test, *p* = 0.2044). (**E**) Latency to feed in NSF in 129 mice. (2-way ANOVA; interaction of PN exposure and adult FLX, F (2, 136) = 3.290, *p = 0.0402; main effect of PN exposure, F (2, 136) = 8.371, ***p = 0.0004, main effect of adult FLX, F (1, 136) = 0.6054, *p* = 0.4379; Bonferroni post-hoc, significant increase in latency to feed with adult FLX treatment in PN-FLX 129 mice, **p = 0.0096). (**F**) Amount of food consumed in the home cage in a 5-min period in 129 mice. (2-way ANOVA; interaction of PN exposure and adult FLX, F (2, 136) = 0.5200, *p* = 0.5957, main effect of PN exposure, F (2, 136) = 0.7566, *p* = 0.4712, main effect of adult FLX, F (1, 136) = 1.356, *p* = 0.2463). Interaction effects from the 2-way ANOVA are represented as significance lines between naïve, PNVEH, and PNFLX 129 groups, and the Bonferroni post-hoc results are depicted as significance lines within the PNFLX 129 group. Light gray squares represent adult vehicle-treated animals. Black circles and checkered bars represent adult fluoxetine-treated animals. 129 s: N = 9 naïve-VEH, 8 naïve-FLX, N = 36 PNVEH-VEH, 32 PNVEH-FLX, 27 PNFLX-VEH, 30 PNFLX-F; C57s: N = 15 PNVEH-VEH, 16 PNVEH-FLX, 17 PNFLX-VEH, 20 PNFLX-FLX. Bars represent mean. Error bars represent standard error. *p < 0.05, **p < 0.01, ***p < 0.001, ****p < 0.0001.

Next, we chronically administered FLX or VEH to PNFLX and PNVEH mice for 3 weeks in their drinking water (Fig. 2A). This method has been shown to decrease avoidant and increase motivated behaviors in the open field and novelty suppressed feeding task (NSF) in C57 mice chronically treated with corticosterone^18^. Mice were retested in the open field. As sex did not significantly interact with adult FLX treatment for any of the variables of interest in PNFLX (2-way ANOVA; 60-min ambulation, F (1, 33) = 1.275, *p* = 0.2670; 10-min rearing, F (1, 33) = 0.0431, *p* = 0.8367; 60-min rearing, F (1, 33) = 1.513, *p* = 0.2273; 10-min center time, F (1, 33) = 2.131, *p* = 0.1538; latency to feed, F (1, 33) = 0.0086, *p* = 0.9266) or PNVEH (2-way ANOVA; 60-min ambulation, F (1, 27) = 0.2850, *p* = 0.5978; 10-min rearing, F (1, 27) = 1.699, *p* = 0.2034; 60-min rearing, F (1, 27) = 0.07306, *p* = 0.7890; 10-min center time, F (1, 27) = 0.0047 *p* = 0.9459; latency to feed, F (1, 27) = 1.324, *p* = 0.2600) mice, we report the data from the sexes combined. Of note, because adult FLX administration is not anxiolytic in unstressed C57 mice^19,25^, we did not perform 2-way ANOVAs to assess if the effects of adult FLX treatment differed by postnatal exposure (PNVEH versus PNFLX) in C57 mice. Instead, for C57 mice we performed unpaired t-tests to examine the effects of adult FLX administration independently within each postnatal exposure group, but we illustrate the data side-by-side in the figures for comparison purposes (Figs. 2 and 3).

In PNFLX mice, we found that chronic FLX administration did not decrease avoidant behavior or increase exploration, and instead exacerbated these behavioral alterations. PNFLX mice administered FLX in adulthood showed decreased rearing (Fig. 2C (left panel); unpaired t-test, *p = 0.0224) and center time in the first 10 min (Fig. 2D (left panel); unpaired t-test, *p = 0.0268; n = 17 PNFLX-VEH, 20 PNFLX-FLX) relative to PNFLX mice given chronic vehicle. As chronic FLX did not change ambulation across the entire 60 min, we believe this reduction in exploratory behavior and increase in avoidant behavior is not due to an overall decrease in locomotor function^26^.

By contrast, chronic FLX administration in PNVEH mice led to an overall decrease in ambulation at 60 min (Fig. 2B (right panel); unpaired t-test, **p = 0.0014) and rearing at both 10 min (Fig. 2C (left panel) unpaired t-test, *p* = 0.0188) and 60 min (Fig. 2C (right panel); unpaired t-test, *p = 0.0103, and did not lead to a significant difference in center time in the first time 10 min (Fig. 2D (left panel); unpaired t-test, *p* = 0.5455; n = 15 PNVEH-VEH, 16 PNVEH-FLX), consistent with prior literature demonstrating that chronic FLX administration leads to decreased locomotion and is not anxiolytic in unstressed C57 mice^19,25^.

Additionally, we tested the PNFLX and PNVEH mice in another measure of avoidant behavior, the NSF. Chronic FLX had no effect on latency to feed in PNFLX (Fig. 3B; unpaired t-test, *p* = 0.6949) or PNVEH mice (Fig. 3B; unpaired t-test, *p* = 0.1314). This was not due to insufficient hunger, as chronic FLX did not affect the amount of food consumed by PNFLX mice in the home cage (Fig. 3C; unpaired t-test, *p* = 0.2393), nor their latency to eat in the home cage (Fig. 3D; unpaired t-test, *p* = 0.2044; n = 17 PNFLX-VEH, 20 PNFLX-FLX). In PNVEH mice, animals treated with FLX in adulthood ate more in their home cage (Fig. 3C; unpaired t-test, *p = 0.0341), even though it did not change latency to eat in the NSF or in the home cage (Fig. 3D; unpaired t-test, *p* = 0.5860; n = 15 PNVEH-VEH, 16 PNVEH-FLX), which are measures more relevant to anxiety.

To determine whether the paradoxical anxiogenic response to chronic FLX in PNFLX mice was the result of early FLX exposure, as opposed to a general result for mice on a C57 background, we examined the effects of chronic adult FLX administration on latency to feed in the NSF in PNFLX, PNVEH, and naïve mice on a 129 background. In the 129 genetic line, adult administration of FLX is known to decrease avoidant behavior even in unstressed control animals^19^, unlike in C57s. We performed a 2-way ANOVA to examine the effects of early postnatal exposure and adult FLX administration and found a significant main effect of postnatal exposure (Fig. 3E; 2-way ANOVA, F (2, 136) = 8.371, ***p = 0.0004) as well as a significant interaction of adult treatment by postnatal exposure in the 129 strain (Fig. 3E; 2-way ANOVA, F(2, 138) = 3.290, *p = 0.0402). Specifically, we found that chronic FLX administration in PNFLX 129 mice in adulthood also increased avoidant behaviors (Fig. 3E; Bonferroni post-hoc, **p = 0.0096). Again, these results were not due to differential effects of hunger as chronic FLX administration did not affect the amount of food consumed in the home cage in any group (Fig. 3F; F (2, 136) = 0.5200, *p* = 0.5957 for interaction between PN exposure and adult treatment, F (2, 136) = 0.7566, *p* = 0.4712 for main effect of PN exposure, F (1, 136) = 1.356, *p* = 0.2463 for main effect of adult FLX).

Cumulatively, these results suggest that rather than being anxiolytic, chronic FLX treatment in adulthood is anxiogenic in mice that received early postnatal FLX exposure.

### Chronic tianeptine decreases avoidant behavior in adult mice postnatally exposed to fluoxetine

**Figure 4:**
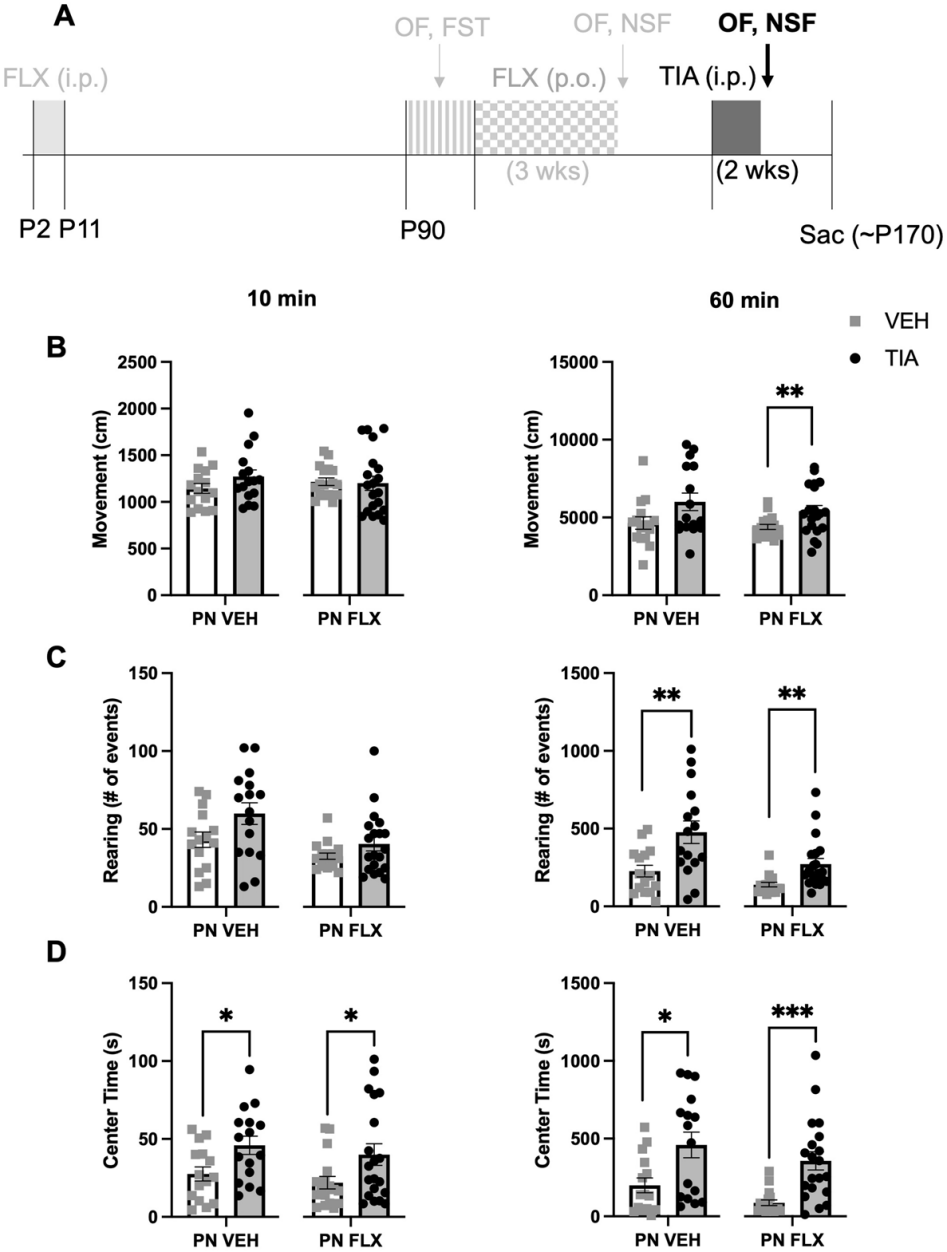
Decreased anxiety-like behavior in the first 10 and full 60 min of open field following adult tianeptine treatment in PNVEH and PNFLX exposed C57 mice. (**A**) Timeline of experiment: The window for developmental administration of fluoxetine as well as prior phases of the experiment are in faded light grey; Black and dark grey indicate tianeptine administration in adulthood and follow up NSF and OF tests. (**B**) Ambulatory movements in the first 10 min of open field (left panel; PNVEH: unpaired t-test, *p* = 0.1643; PNFLX: unpaired t-test, *p* = 0.8569). Same for total 60 min (right panel; PNVEH: unpaired t-test, *p* = 0.0594; PNFLX: unpaired t-test, **p = 0.0096) (**C**) Rearing movements in the first 10 min (left panel, PNVEH: unpaired t-test, *p* = 0.0631; PNFLX: unpaired t-test, *p* = 0.1405). For total 60 min (right panel; PNVEH: unpaired t-test, **p = 0.0053; PNFLX: unpaired t-test, **p = 0.0057) (**D**) Time spent in center in the first 10 min (left panel, PNVEH: unpaired t-test, *p = 0.0198; PNFLX: unpaired t-test, *p = 0.0386). For total 60 min (right panel, PNVEH: unpaired t-test, *p = 0.0120; PNFLX: unpaired t-test, ***p = 0.0002). Light gray squares represent adult vehicle-treated animals. Black circles and grey bars represent adult tianeptine-treated animals. N = 15 PNVEH-VEH, 16 PNVEH-TIA, 17 PNFLX-VEH, 20 PNFLX-TIA. Bars represent mean. Error bars represent standard error. *p < 0.05, **p < 0.01, ***p < 0.001, ****p < 0.0001.

**Figure 5:**
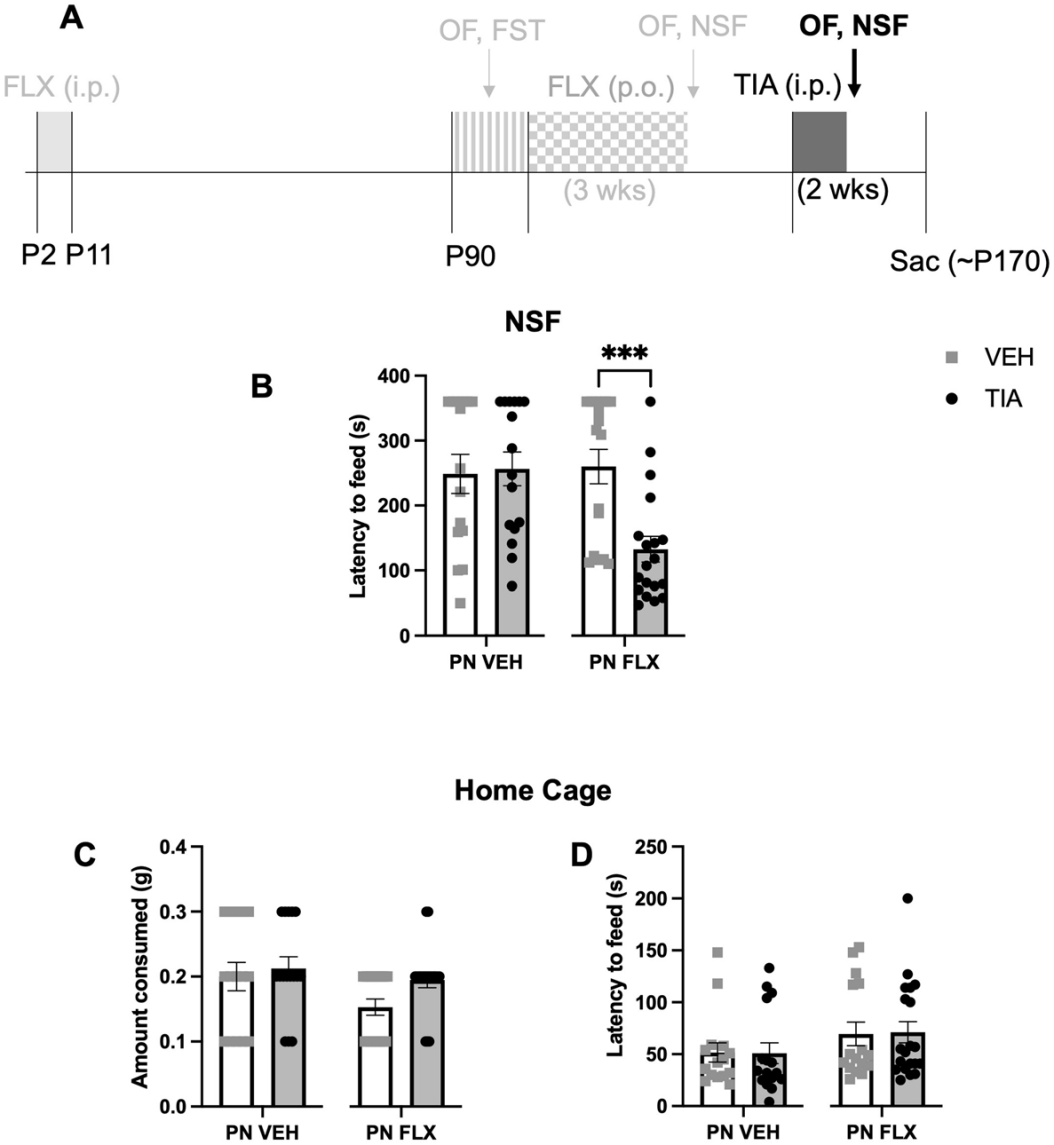
Decreased anxiety-like behavior in the novelty suppressed feeding task following adult tianeptine treatment in C57 PNFLX mice. (**A**) Timeline of experiment: The window for developmental administration of fluoxetine as well as prior phases of the experiment are in faded light grey; Black and dark grey indicate tianeptine administration in adulthood and follow up NSF and OF tests. (**B**) Latency to feed in NSF in C57 mice (PNVEH: unpaired t-test, *p* = 0.8455; PNFLX: unpaired t-test, ***p = 0.0004). (**C**) Amount of food consumed in the home cage in a 5-min period in C57 mice (PNVEH: unpaired t-test, *p* = 0.6599; PNFLX: unpaired t-test, *p* = 0.0679). (**D**) Latency to eat in the home cage in C57 mice (PNVEH: unpaired t-test, *p* = 0.7460; PNFLX: unpaired t-test, *p* = 0.9435). Light gray squares represent adult vehicle-treated animals. Black circles and grey bars represent adult tianeptine-treated animals. N = 15 PNVEH-VEH, 16 PNVEH-TIA, 17 PNFLX-VEH, 19 PNFLX-TIA. Bars represent mean. Error bars represent standard error. *p < 0.05, **p < 0.01, ***p < 0.001, ****p < 0.0001.

Following a two-week washout period, C57 PNFLX and PNVEH mice were pseudorandomly assigned into chronic TIA and VEH groups and administered the drug for 14 days (Fig. 4A). A twice-per-day dose of 30 mg/kg was previously shown to normalize avoidant, motivated, and hedonic behaviors in mice chronically treated with corticosterone^14^. Recent data from our lab has shown that administration for an even shorter time course of 7 days is sufficient to see these behavioral changes^27^. As sex did not significantly interact with adult TIA treatment for any of the variables of interest in PNFLX mice (2-way ANOVA; 60 min ambulation, F (1, 33) = 1.879, *p* = 0.21797; 10 min rearing, F (1, 33) = 1.904, *p* = 0.1769, 4; 60 min rearing, F (1, 33) = 0.8699, *p* = 0.3578; 10 min center time, F (1, 33) = 0.1551, *p* = 0.6963; latency to feed, F (1, 33) = 2.017, *p* = 0.1652) or PNVEH (2-way ANOVA; 60 min ambulation, F (1, 27) = 0.0589, *p* = 0.8099; 10 min rearing, F (1, 27) = 0.8085, *p* = 0.3765; 60 min rearing, F (1, 27) = 0.3828, *p* = 0.5413; 10 min center time, F (1, 27) = 0.1288, *p* = 0.7224; latency to feed, F (1, 27) = 1.462, *p* = 0.2371), we report the data from the sexes combined. To test whether tianeptine is effective at decreasing avoidant behavior and normalizing exploratory behavior in PNFLX exposed animals, we focused on comparing the tianeptine treated versus vehicle-treated PNFLX group using unpaired t-tests, but report the data from PNVEH mice side-by-side for comparison purposes (Figs. 4 and 5).

In PNFLX mice, chronic TIA significantly increased rearing at 60 min (Fig. 4C (right panel); unpaired t-test, **p = 0.0057), as well as center time in the first 10 min (Fig. 4D (left panel); unpaired t-test, *p = 0.0386) and the entire 60 min (Fig. 4D (right panel); unpaired t-test, ***p = 0.0002). In these mice, chronic TIA administration had no effect on rearing (Fig. 4C (left panel); unpaired t-test, *p* = 0.1405) or ambulation in the first 10 min of the OF (Fig. 4B (left panel); unpaired t-test, *p* = 0.8569). However, there was a significant increase in 60-min ambulation (Fig. 4B (right panel); unpaired t-test, **p = 0.0096; n = 17 PNFLX-VEH, 20 PNFLX-TIA). These findings suggest that chronic TIA administration increases novelty-induced exploration, and decreases avoidant behavior, in PNFLX mice, but that some of these effects may be mediated by boosting overall movement.

To evaluate if TIA would induce the same increase in exploration and locomotion in PNVEH animals, we looked at the effect of chronic adult treatment with TIA versus VEH in this group. We see that in PNVEH mice, chronic TIA resulted in the same significant increase in rearing at 60 min (Fig. 4C (right panel); unpaired t-test, **p = 0.0053), center time in the first 10 min (Fig. 4D (left panel); unpaired t-test, *p = 0.0198) and the entire 60 min (Fig. 4D (right panel); unpaired t-test, *p = 0.0120). PNVEH-TIA mice also showed no change in ambulation in the first 10 min of the OF (Fig. 4B (left panel); unpaired t-test, *p* = 0.1643). Unlike the PNFLX mice, chronic TIA administration in adulthood in PNVEH mice resulted in a trend towards increased rearing in the first 10 min (Fig. 4C (left panel); unpaired t-test, *p* = 0.0631) and a trend towards a significant increase in 60-min ambulation (Fig. 4B (right panel); unpaired t-test, *p* = 0.0594; n = 15 PNVEH-VEH, 16 PNVEH-TIA). Thus, chronic TIA appears to reduce avoidant behavior, and boost overall movement, in PNVEH mice, similar to PNFLX mice.

We also examined whether chronic TIA would improve avoidant behaviors in the NSF in PNFLX mice, as a separate measure of avoidant behavior that would not be as influenced by changes in overall locomotion. One animal from the PNFLX-TIA group was excluded due to an error in collecting control home cage measurements. Chronic TIA treatment in PNFLX mice decreased latency to feed in the NSF when compared to PNFLX-VEH mice (Fig. 5B; unpaired t-test, ***p = 0.0004) TIA had no effect on home cage food consumption or latency to feed in PNFLX mice (Fig. 5C; unpaired t-test, *p* = 0.0679; Fig. 5D; unpaired t-test, *p* = 0.9435; n = 17 PNFLX-VEH, 19 PNFLX-TIA), confirming that the decreased latency to feed was not due to a change in hunger. Chronic administration with TIA in PNVEH mice did not alter their latency to feed in the NSF (Fig. 5B; unpaired t-test, *p* = 0.8455), nor their consumption or latency in their home cage (Fig. 5C; amount consumed: unpaired t-test, *p* = 0.6599; Fig. 5D; latency in the home cage: unpaired t-test, *p* = 0.7460; n = 15 PNVEH-VEH, 16 PNVEH-TIA), suggesting that TIA is primarily effective at reducing avoidant behavior in the NSF in PNFLX, but not PNVEH, mice. Cumulatively, these data suggest that, unlike chronic FLX, chronic TIA decreases avoidant symptoms in adult PNFLX mice.

Because of the crossover nature of these experiments, we verified that prior administration with chronic FLX or vehicle did not impact the effect of TIA in open field test avoidant behaviors (Fig. S1; B (left-panel), rearing in first 10 min, two-way ANOVA, F(1, 33) = 1.522, *p* = 0.2260, no interaction of adult FLX and TIA administrations; B (right panel), rearing in 60 min, two-way ANOVA, F(1, 33) = 0.0491, *p* = 0.8259, interaction of adult FLX and TIA administrations; C (left panel), center time in first 10 min, two-way ANOVA, F(1, 33) = 0.3396, *p* = 0.5640, no interaction of adult FLX and TIA administrations; C (right panel) center time in 60 min, two-way ANOVA, F(1, 33) = 0.004, *p* = 0.9479, no interaction of adult FLX and TIA administrations) or the NSF (two-way ANOVA, F(1, 32) = 0.3416, *p* = 0.5630, no interaction of adult FLX and TIA administrations) in PNFLX mice.

We did find interaction effects between the adult treatments specifically in PNVEH animals. We observed that prior exposure to fluoxetine in adulthood blunted the increased movement observed with TIA treatment in PNVEH animals in the 10-min movement (Fig. S2A (left panel); 2-way ANOVA, left panel, F(1, 27) = 7.242, *p = 0.0121, interaction of adult FLX and TIA administration; Bonferroni post-hoc, significant increase in movement in PNVEH-VEH-TIA animals relative to PNVEH-VEH-VEH animals, *p = 0.0112), 60-min movement (Fig. S2A (right panel); 2-way ANOVA, right panel, F (1, 27) = 8.553, **p = 0.0069, interaction of adult FLX and TIA administrations; Bonferroni post-hoc, significant increase in movement in PNVEH-VEH-TIA animals relative to PNVEH-VEH-VEH animals, **p = 0.0016), and 60-min rearing (Fig. S2B (right panel); 2-way ANOVA, F (1, 27) = 5.968, *p = 0.0214, interaction of adult FLX and TIA administrations; Bonferroni post-hoc, significant increase in rearing in PNVEH-VEH-TIA animals relative to PNVEH-VEH-VEH animals, ***p = 0.0002) measurements. However, as measures of avoidant behavior were not significantly different between animals administered VEH or FLX in adulthood (10-min center time: Fig. S2C (left panel); 2-way ANOVA, F (1, 27) = 0.4751, *p* = 0.4965), this implies a more general impact of fluoxetine exposure on locomotion, aligning with previous work showing decreased ambulation following fluoxetine administration in unstressed C57 males and females^28^.

## Discussion

### Avoidant behavior following early fluoxetine exposure

We found that PNFLX C57 mice showed decreased novelty-induced exploration and increased avoidant behavior, similar to effects seen in PNFLX 129 mice. However, PNFLX C57 mice did not show the same increases in behavioral despair seen in PNFLX 129 animals. This strain difference is consistent with other developmental SSRI studies in mice. For example, C57 mice treated with FLX from P4–P21 showed reduced distance traveled and time spent moving in the open field^24,29^. Similarly, C57 offspring gestationally exposed to FLX from E8-E18, showed an increased latency to feed in the NSF test as adults^30^. Moreover, these strain differences are consistent with previous work demonstrating differing behavioral effects of deleting the serotonin transporter (5-HTT-KO) in mice of different genetic backgrounds. While 5-HTT-KOs on a C57 background showed more avoidant behavior, those on a 129 background showed more pronounced behavioral despair^31^. Cumulatively, comparing the effects of developmental SSRI exposure in different strains of mice contributes to our understanding of how genetic background can interact with environmental exposures to influence the presentation of behavioral symptoms associated with mood and anxiety disorders^7^.

### Adult fluoxetine administration increases avoidant behavior in PNFLX mice

Intriguingly, chronic FLX administration did not improve, and even exacerbated, affective symptoms in PNFLX mice. These changes included decreased novelty-induced exploration and increased avoidant behaviors. The paradoxical effects of chronic FLX administration on avoidant behavior in adult PNFLX mice could occur because the serotonergic system is persistently disrupted in these mice. Early postnatal fluoxetine exposure produces adult mice with a hyperactive median raphe, hypoactive dorsal raphe^32^, and altered prefrontal modulation of both serotonergic and non-serotonergic raphe neurons^33^. When serotonergic activity was pharmacologically inhibited in the median raphe in PNFLX mice, avoidant behavior was decreased, implying that hyperactivity in the median raphe plays a key role in regulating this behavior in PNFLX animals^32^. Adult SSRI administration may be ineffective at relieving, and possibly aggravates avoidant, anhedonic, and motivation-related behaviors because of this different baseline set-point of serotonergic neurons in PNFLX mice.

One concern was that our C57 PNFLX mice were not ‘stressed’ enough for chronic FLX administration to decrease avoidant behavior^17,19^. However, even on a 129 background where PNFLX mice have a very pronounced affective behavioral phenotype^8,9^, chronic adult FLX administration paradoxically increased avoidant behavior, an effect that was not seen in PNVEH mice. These findings in both C57 and 129 PNFLX mice indicate that postnatal FLX exposure unexpectedly either has no effect or increases avoidant behavior in response to adult FLX administration.

Our findings differ from those of Karpova et al., who found that adult chronic FLX exposure improved several measures of avoidant behavior in mice postnatally treated with FLX from P4–P21^24^. Although in 129 mice P2–P11 and P4–P21 postnatal FLX administration produce many of the same adult behavioral differences, Rebello et al. showed that in the elevated plus maze, the effects of P2–P11 and P12–P24 PNFLX are actually diametrically opposed, with P2–P11 being anxiogenic and P12–P24 being anxiolytic^8^. Thus, differences in the length of postnatal FLX exposure may explain the differences in our findings. As the P2–P11 window better approximates the brain development occurring during the third trimester of a human pregnancy, we believe our results might be more applicable to the human condition.

### Adult tianeptine administration decreases avoidant behavior in PNFLX mice

In contrast to chronic FLX, chronic TIA administration increased novelty-induced exploratory behavior and motivation, and decreased avoidant behavior in adult PNFLX mice. Surprisingly, TIA also increased ambulation in the open field test, an effect that is notable as it should not result from a direct psychostimulant effect of the drug, which was administered 14 h prior to all behavioral tests and TIA clears from both blood plasma and brain tissue within 2 h^14^. Interestingly, TIA also increased exploratory and ambulatory, and decreased avoidant, behavior in the open field in PNVEH animals. However, in the NSF, the ability of TIA to decrease latency to feed was specific to the PNFLX, but not PNVEH, animals. Importantly, TIA did not impact latency to feed in the home cage in either group of mice, suggesting that these results are not a result of increased hunger. The differential efficacy of TIA in PNFLX, but not PNVEH, mice in the NSF may reflect that the drug is specifically anxiolytic in PNFLX mice. However, given the confounding pro-ambulatory effects seen in both PNFLX and PNVEH animals in the open field test, the anxiolytic effect of TIA in PNFLX animals may not generalize to all contexts.

How chronic TIA decreases avoidant behaviors in PNFLX mice, while chronic FLX does not, remains an important open question. One possible reason for the difference in efficacy is due to persistent alterations in the serotonergic system of PNFLX mice preventing the typical mechanism of action of FLX administration. Recent work has revealed that PNFLX exposure results in decreased glutamate EPSCs when 5-HT inputs onto DA neurons are stimulated in vitro^34^. FLX’s proposed mechanism of action involves blocking serotonin reuptake, ultimately increasing monoamine levels over time and leading to enhanced plasticity and hippocampal neurogenesis, which also contribute to the drug’s behavioral efficacy^5^.

By contrast, TIA does not act at  serotonin receptors/transporters, unlike SSRIs. TIA also does not directly target other monoaminergic systems but instead has been shown to be a MOR agonist^13,35^. MORs are necessary for the improvement in affective symptoms elicited by chronic administration of TIA in mice chronically administered corticosterone^14^, while acute depletion of serotonin does not affect TIA’s efficacy (Rene Hen, personal communication). TIA provokes mesolimbic dopamine release, despite having little to no affinity for DA receptors/transporters, and does this in a serotonin independent manner^36^. Interestingly, it also increases the responsivity of the α1-andrenergic system^37^. TIA does not inhibit biogenic amine transporters but has been shown to indirectly modulate glutamatergic transmission and promote neuroplasticity^35,38^. These differences may explain the differential behavioral efficacy of these two drugs in PNFLX mice.

In addition, the Ansorge lab has shown postnatal FLX exposure leads to reduction in medial prefrontal cortex (mPFC) dendritic branching in layer 2–3 pyramidal cells, an effect also seen in early life stress models of depression in rodents^8^. As treatment with TIA (but not FLX) prevents atrophy of apical dendritic branches in the CA3 of the hippocampus ^39^ following stress, TIA may similarly promote regrowth of mPFC dendritic branches in PNFLX animals, a possibility that remains of interest to ourselves and others.

Another possibility for TIA’s mechanism of action in PNFLX mice is through targeting MORs in the habenula, a region that has been previously implicated in depression^40–42^. The habenula has the densest MOR population in the brain and has inhibitory projections to major regulators of emotional behavior, like the VTA. The region has been shown to be hyperactive in MDD patients^43^, and activation of the habenula is correlated with decreased VTA DA release. Tianeptine may function in part by activating inhibitory MORs in the habenula, thereby disinhibiting downstream regions like the VTA^44–46^. Also, recent work has shown inhibition of DRN 5-HT neurons rescued changes in emotional behavior in PNFLX animals^32^, and that inhibition of DRN 5HT neuronal projections to the habenula decreased habenula activity and reduced depressive behavior^40^. Tianeptine may mimic this effect, as MOR activation is typically inhibitory and would also decrease habenula activity, without directly targeting the serotonin system.

### Limitations

In our experiment, we were conscientious of how models of affective behavior and treatment often have differential effects in males and females. As we did not find an interaction of adult FLX or TIA treatment by sex, all our subsequent analysis is shown with the sexes combined.

The same cohort of PNFLX or PNVEH animals first underwent chronic administration with FLX or its respective vehicle, followed by chronic administration with either TIA or its respective vehicle. While it is possible that the first drug administration could have influenced the outcome of the second, we believe this is unlikely as animals were randomly reassigned to treatment or vehicle conditions and allowed a two-week washout period between the chronic administrations. We did not observe an effect of crossover using direct statistical assessment of any measure in PNFLX mice but did find interactions in PNVEH mice in overall 60-min movement (Fig. S2A (right panel); 2-way ANOVA, F (1, 27) = 8.553, **p = 0.0069, interaction of adult FLX and TIA administrations; Bonferroni post-hoc, significant increase in movement in PNVEH-VEH-TIA animals relative to PNVEH-VEH-VEH animals, **p = 0.0016). As we see a marked increase in overall movement and no interaction in 10-min center time, prior exposure to fluoxetine may blunt the locomotor effects of chronic tianeptine, as opposed to effecting measures of anxiety. Importantly, this repeated administration paradigm—first with an SSRI followed by another drug—most closely matches the experience of SSRI-resistant patients.

### Broader implications

The goal of precision medicine is to identify the most effective treatment for a given patient based on known risk factors, biomarkers, or specific genes^47^. Here we show resistance to adult FLX administration in a developmental FLX exposure model. Future studies should examine whether similar exposure to other SSRIs, such as escitalopram, paroxetine, or sertraline would also have these effects. Furthermore, the efficacy of TIA at normalizing avoidant behavior in this PNFLX model suggests non-monoaminergic targeting therapeutics may be most efficacious in persons with a history of this early in utero exposure. More broadly, non-monoamine antidepressants like TIA may also be more efficacious in other models of treatment resistance.

In summary, here we show that TIA may be a promising alternative treatment from SSRIs for humans with suspected in utero early developmental exposure to SSRIs, and more broadly that it may be helpful in a subset of SSRI-resistant depression.

## Supplementary Information

Supplementary Information 1.

Supplementary Information 2.
