# Supplementary figures and images for: Tianeptine, but not fluoxetine, decreases avoidant behavior in a mouse model of early developmental exposure to fluoxetine
Source: Sci Rep. 2021 Nov 24;11:22852. doi: 10.1038/s41598-021-02074-9 (PMC8613176; doi:10.1038/s41598-021-02074-9)

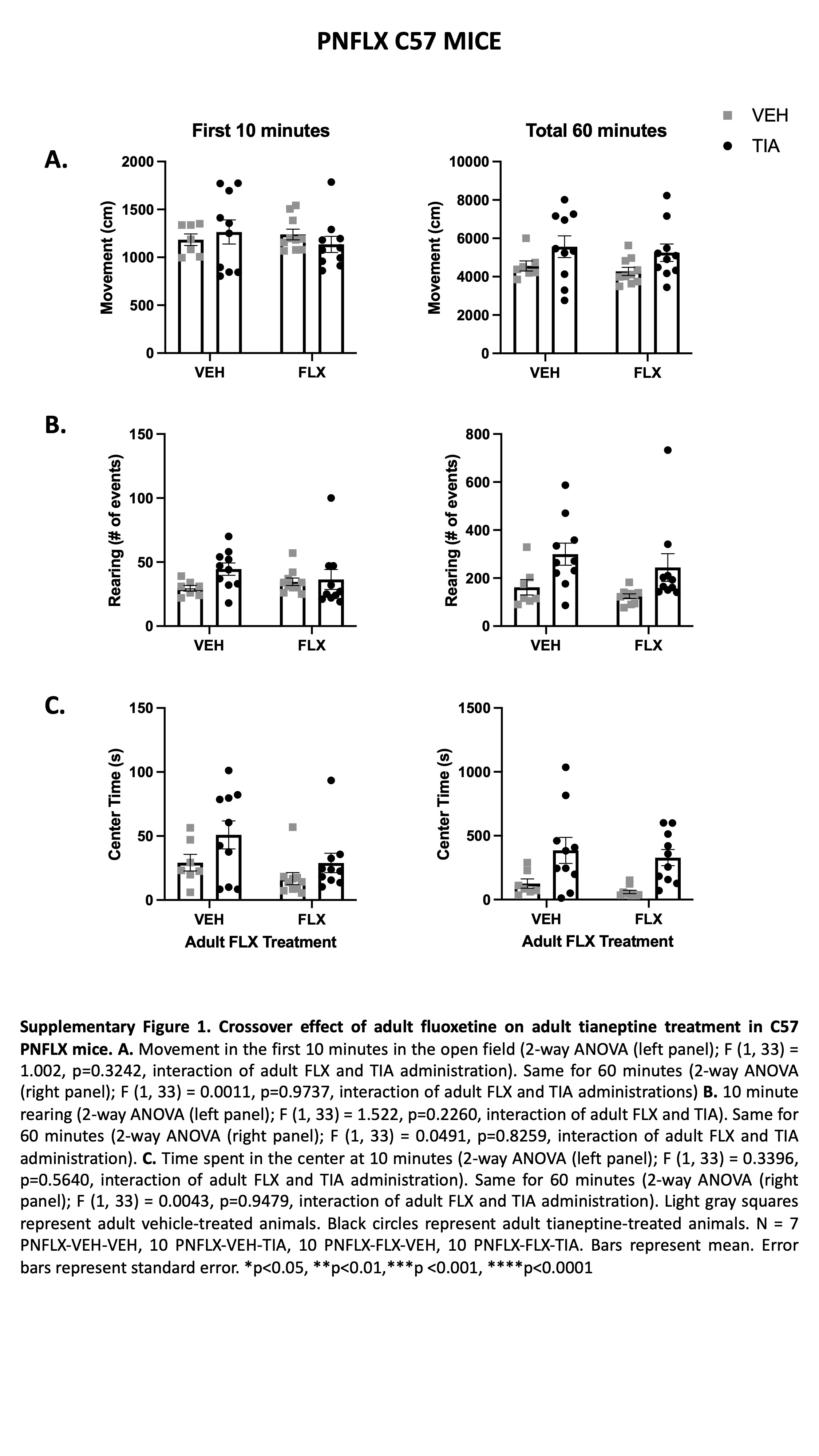

Supplement: Supplementary file 1 — Supplementary Information 1. [file 41598_2021_2074_MOESM1_ESM.jpg]

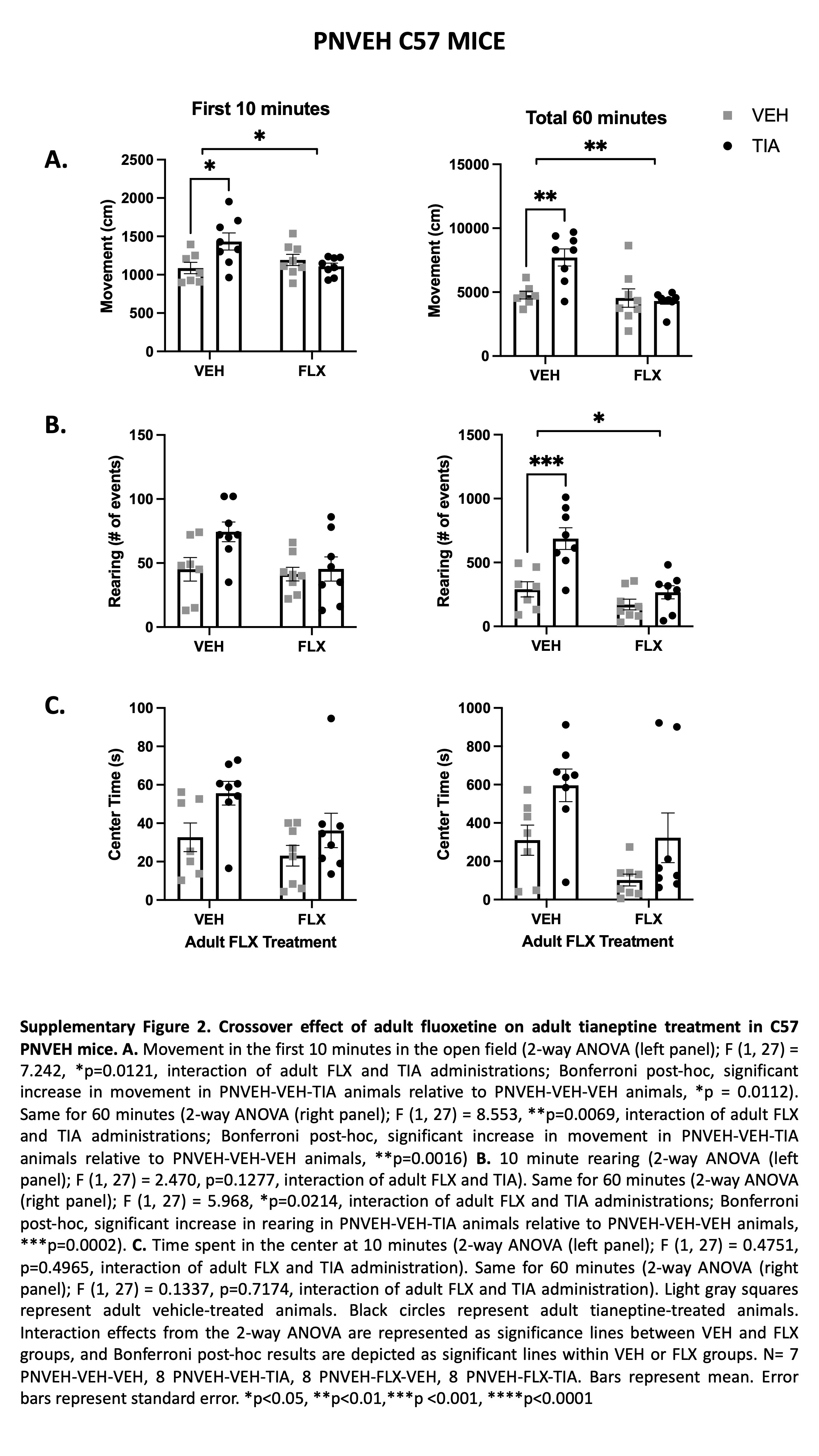

Supplement: Supplementary file 2 — Supplementary Information 2. [file 41598_2021_2074_MOESM2_ESM.jpg]
